# Thoracoscopic McKeown esophagectomy in a patient with an azygos lobe

**DOI:** 10.1186/s13019-024-02621-1

**Published:** 2024-03-15

**Authors:** Rie Nakashima, Kohei Tajima, Kazuo Koyanagi, Akihito Kazuno, Miho Yamamoto, Yoshiaki Shoji, Kentaro Yatabe, Kohei Kanamori, Mika Ogimi, Kazuhito Nabeshima, Kenji Nakamura, Masaki Mori

**Affiliations:** Department of Gastroenterological Surgery, Tokai University School of Medicine, 143 Shimokasuya, Isehara, Kanagawa 259-1193 Japan

**Keywords:** Azygos lobe, Esophageal carcinoma, McKeown esophagectomy, Thoracoscopy, Anatomical variation

## Abstract

**Background:**

The azygos lobe is a relatively rare anatomical variation, and there have been no reports, until date, of thoracoscopic McKeown esophagectomy for esophageal cancer in a patient with an azygos lobe. The azygos lobe can be diagnosed by chest X-ray or CT, and is usually not associated with any symptoms. However, surgeons should be aware that transthoracic surgical procedures in patients with an azygos lobe could be associated with a high risk of complications.

**Case presentation:**

An 83-years-old man was brought to our emergency room with fever, severe headache, and difficulty in moving. MRI revealed a brain abscess, which was treated by abscess drainage and systemic antibiotic treatment. Further examinations to determine the cause of the brain abscess revealed esophageal cancer. In addition, CT revealed an azygos lobe in the right thoracic cavity. Although intrathoracic adhesions were anticipated on account of a previous history of bacterial pyothorax, we decided to perform esophagectomy via a thoracoscopic approach. Despite the difficulty in dissecting the intrathoracic adhesions, we were able to obtain the surgical field thoracoscopically. Then, we found the azygos lobe, as diagnosed preoperatively, and the azygos vein was supported by the mesentery draining into the superior vena cava. After dividing the mesentery, we clipped and cut the vessel, and both ends were further ligated. After these procedures, we safely performed esophagectomy with 3-field lymph node dissection. The postoperative course was uneventful, and the patient was discharged on the 21st postoperative day.

**Conclusions:**

Although there was a firm adhesion in the thoracic cavity, preoperative recognition of the azygos lobe could help in preventing intraoperative injury. Especially, esophageal surgeons are required to deal with the azygos lobe safely to avoid serious intraoperative injury.

## Background

The azygous lobe is a rare anatomical variation, reported to be 0.30% in a meta-analysis [1]. There have been only a few reports of thoracoscopic esophagectomy for esophageal cancer patient coexisting azygos lobe. Herein, we report a case in which esophageal cancer in a patient with an azygos lobe was treated successfully and safely by thoracoscopic surgery.

## Case presentation

### History of present illness

**Fig. 1:**
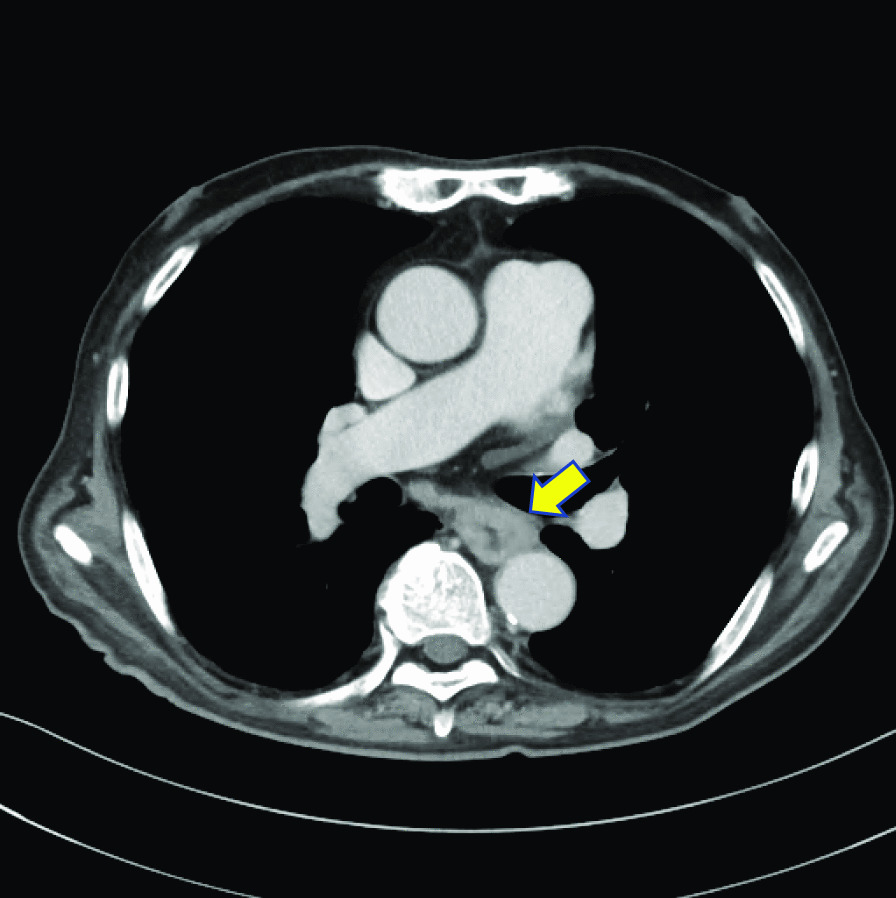
Enhanced CT examination demonstrating wall thickening with contrast effect in the middle thoracic esophagus (arrow)

An 83-year-old man was brought to our emergency room with 10-day history of fever, severe headache, and difficulty in moving. MRI revealed a brain abscess, which was treated by abscess drainage and systemic antibiotic treatment. Although the patient had no symptoms, a CT performed to determine the cause of the brain abscess revealed thickening of the wall of the esophagus (Fig. 1), and the patient was referred to our department.

**Fig. 2:**
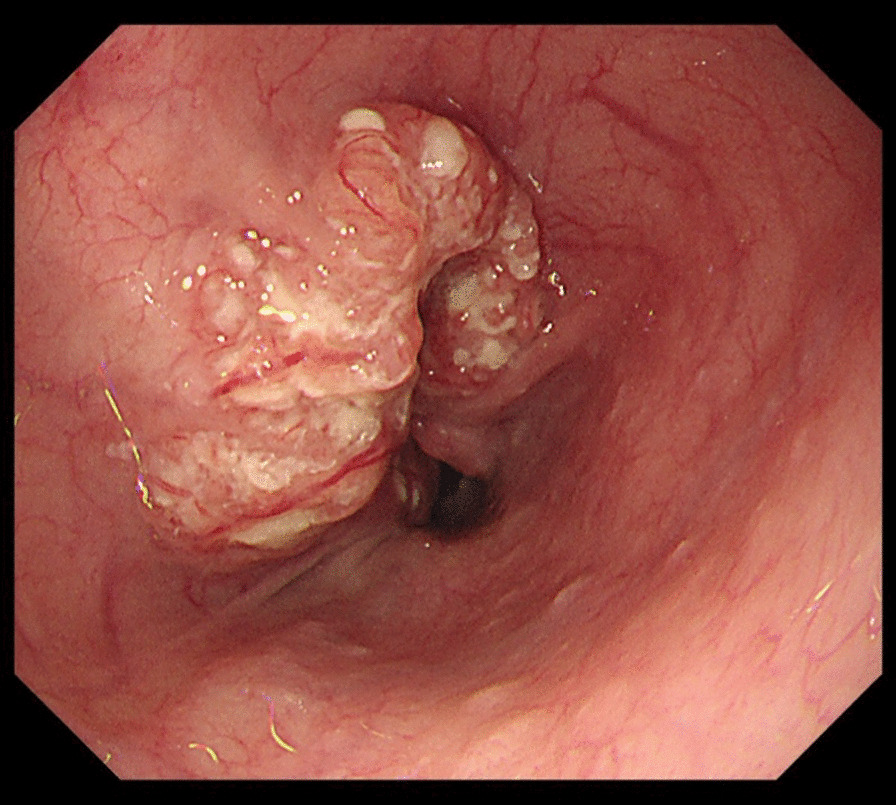
A Type 2 lesion measuring 6 cm in length was detected in the middle thoracic esophagus

**Fig. 3:**
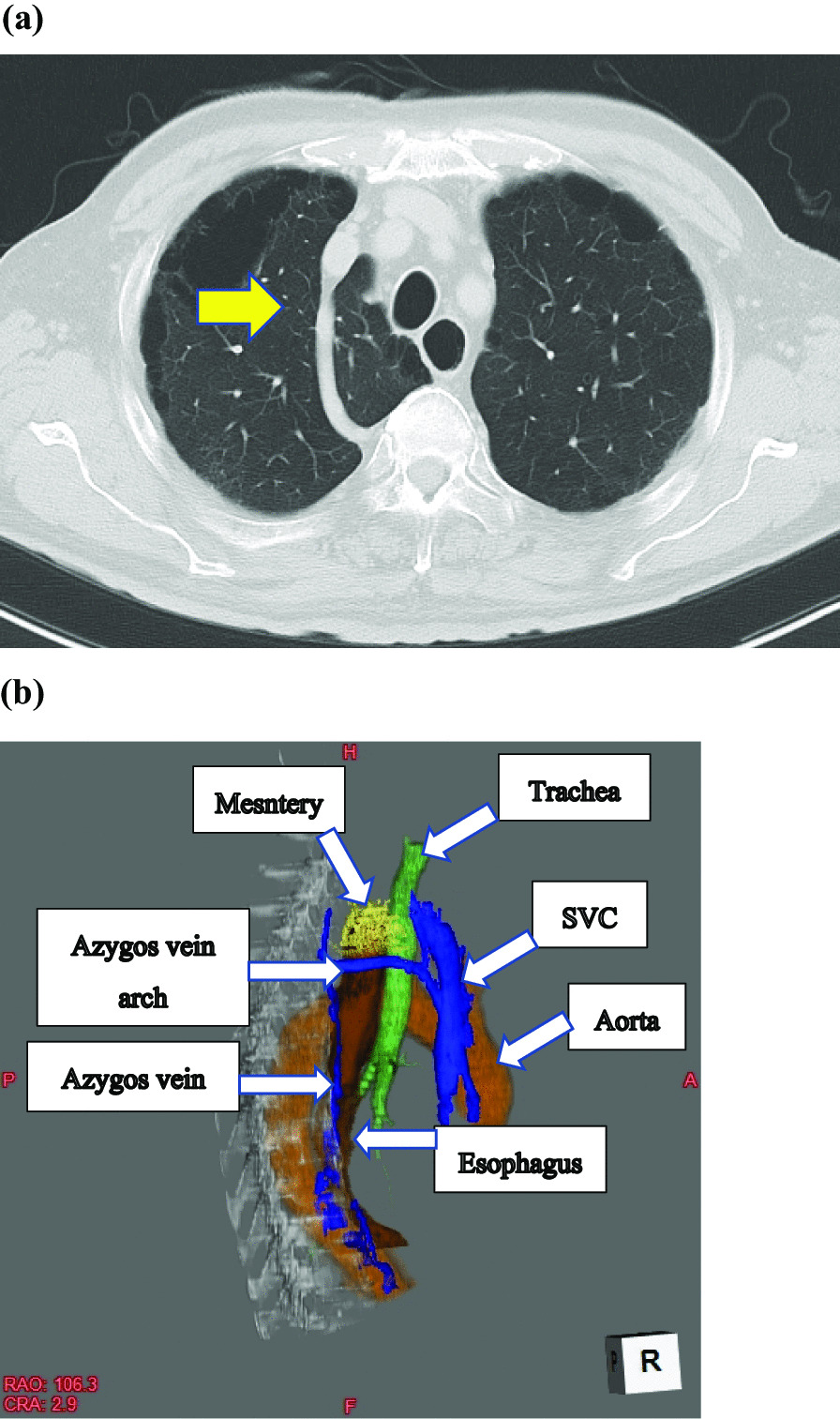
Azygos lobe. Axial image of CT (arrow) **(a)**, and 3D-CT **(b)**. SVC: superior vena cava

Barium swallow revealed a tumor measuring 6 cm in length with an abnormal ulcer mound in the middle thoracic esophagus; endoscopy revealed a Type 2 tumor (Fig. 2). Histopathological examination of biopsy specimens revealed the squamous cell carcinoma. Abdominal and chest CT revealed wall thickening in the middle thoracic esophagus without invasion of the adjacent mediastinal organs or mediastinal lymph node metastasis. In addition, CT revealed an azygos lobe in the right thoracic cavity (Fig. 3a, b). We planned the esophagectomy for the esophageal cancer after control of the brain abscess. Although intrathoracic adhesions were anticipated on account of a previous history of bacterial pyothorax 14 years ago, we decided to perform esophagectomy via a thoracoscopic approach without any preoperative treatment because of his advanced age. After consulting with the respiratory surgeons, we planned to resect the abnormal azygos vein during esophagectomy.

### Surgical procedure

**Fig. 4:**
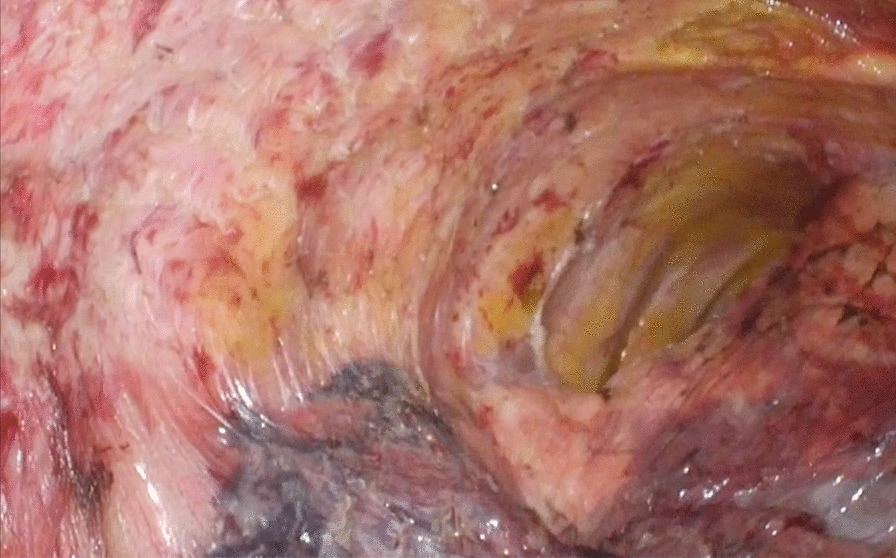
There was strong adhesions around the esophagus in the middle and lower mediastinum

**Fig. 5:**
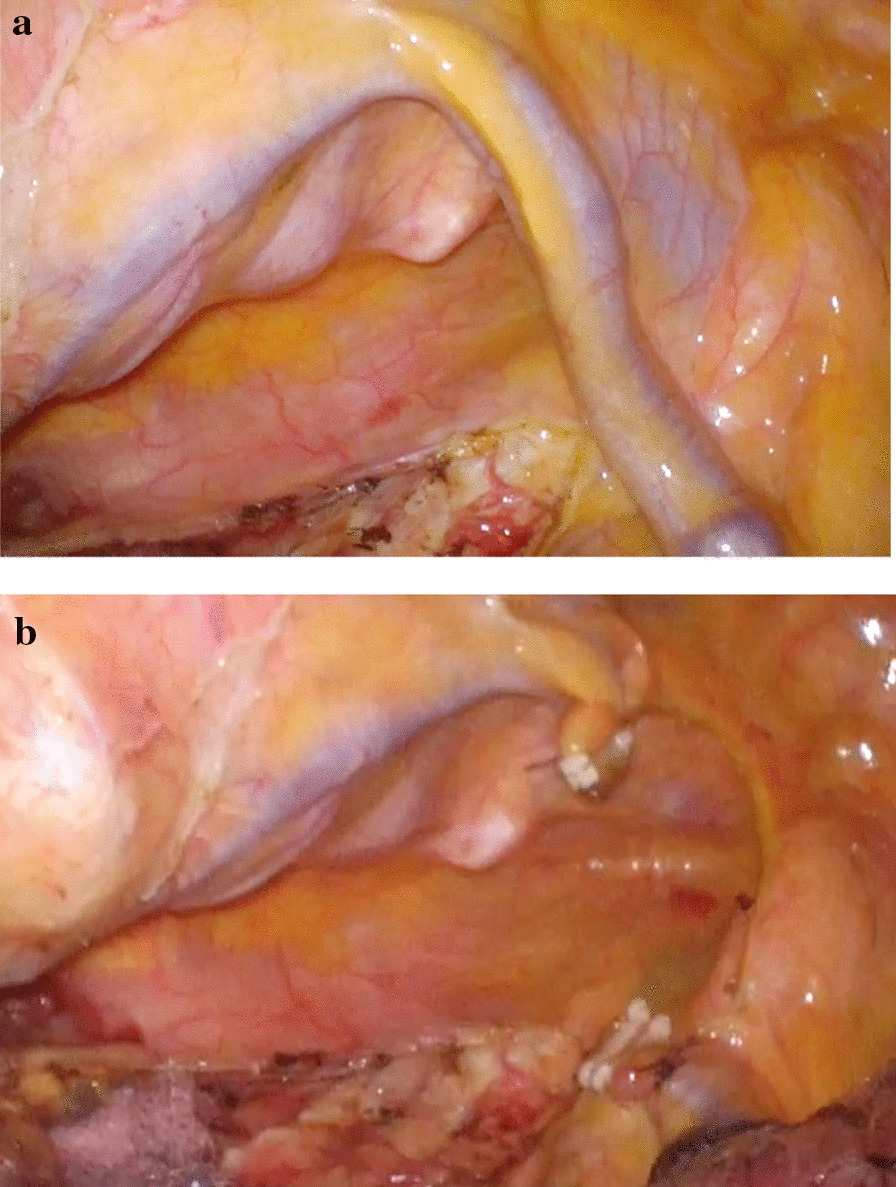
The azygos vein was present in the thoracic cavity, supported by the mesentery, and flowed into the superior vena cava **(a)**. The azygos vein was clipped and cut with a vessel-sealing system, and both ends were ligated using the endloop™ **(b)**

The patient was placed in the prone position. Due to the adhesions in the thoracic cavity as expected, insertion of the trocars required some ingenuity. We performed dissection of adhesions using a 10-mm flexible scope and obtained an adequate field of view. Trocars were inserted as needed while performing dissection of adhesion, and we finally inserted the four trocars into the right thoracic cavity: three 12-mm trocars into the fifth intercostal space on the posterior axillary line, seventh intercostal space at the midpoint between the inferior scapular angle line and the posterior axillary line, and ninth intercostal space on the level of the inferior scapular angle, and a 5-mm trocar into the sixth intercostal space on the mid-axillary line. Although we usually insert a trocar into the third intercostal space on the mid-axillary line, we could not insert it due to adhesion of upper lobe of the of right lung. Therefore, we performed all surgical procedures via the four trocars. Then, we continued the dissection of remaining adhesion in the thoracic cavity. The middle and lower mediastinum was manipulated first because of the strong adhesions around the esophagus in the middle and lower mediastinum and the tumor's extensive contact with the left main bronchus on preoperative CT (Fig. 4). Fortunately, the adhesions were detached without any damage, and the esophagus could be dissected from surrounding organs. Adhesions around the upper esophagus were not severe and fortunately found the azygos lobe easier than expected, and the azygos vein was supported by the mesentery draining into the superior vena cava (Fig. 5a). It might interfere with forceps operation and the surgical field or pose a risk of injury during esophagectomy, after dividing the mesentery, we clipped and cut the vessel with a vessel-sealing system, and both ends were ligated using the endloop™ (Fig. 5b). After that, we performed McKeown esophagectomy with dissection for three field lymph nodes including around bilateral recurrent laryngeal nerve, as previously described [2]. The operation time for the thoracic part was 325 min, and the blood loss during the thoracic part of the surgery amounted to 29 ml. The postoperative course was uneventful and physical functions that were impaired by the brain abscess recovered well. The patient was discharged on the 21st postoperative day. Histopathological examination of the resected specimen confirmed the diagnosis of esophageal squamous cell carcinoma, and the lesion was classified as pT3, pN0, M0, pStageIIB (UICC 8th).

## Discussion and conclusion

The azygos lobe was first recognized at autopsy in 1877 [3]. It is found in 1.0% of all anatomic specimens and about 0.4% of all chest radiographs. In the 2021 literature, a random-effects meta-analysis reported a overall prevalence of 0.30% [1]. Azygos lobe is formed when the right posterior cardinal vein, one of the precursors of the azygos vein, penetrates the apex of the lung instead of migrating over it during embryogenesis. As developmental changes occur, at about the level of the fourth thoracic vertebra, the azygos vein arches anteriorly and to the left, passes over the right upper lobe bronchus, and then enters the posterior surface of the superior vena cava. The azygos vein lies within the thoracic cavity and a veil, a mesentery-like structure derived from the mediastinal pleura, separates the medial side of the right upper lobe to from the azygos lobe [3, 4].

Patients with an azygos lobe are usually asymptomatic, and do not present a clinical problem. However, it has been reported that rarely, compression of the azygos arch could cause flexion of the azygos lobe bronchus, resulting in atelectasis, bronchiectasis, and pulmonary infections [5]. At the time of this examination, our patient showed no symptoms, and the azygos lobe was found incidentally on CT performed to determine the clinical stage of esophageal cancer in the patient. However, it may be possible that intrathoracic adhesion might be associated with pulmonary infection due to the azygos lobe [6].

The azygos lobe can be diagnosed by chest X-ray or, especially CT [7, 8]. Presence of an azygos lobe can be detected on CT, as in our case, by a thickened fissure running from the posterior vertebral body to the superior vena cava [8]. The azygos lobe may be confused with a bulla or abscess, or a pulmonary nodule [9]. It is important to be aware of the radiological features of an azygos lobe to avoid misinterpretation; according to one survey, most clinical residents at one institution were unaware of the azygos lobe [10].

**Fig. 6:**
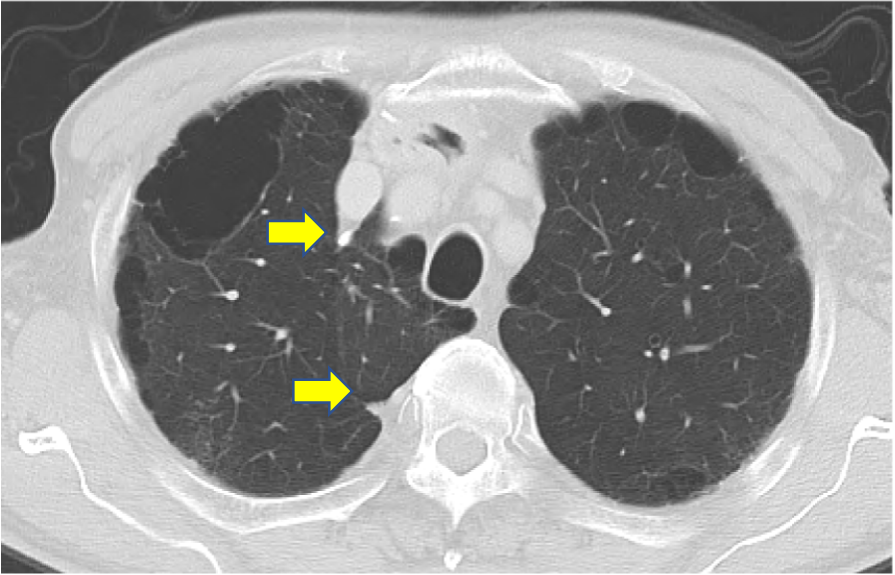
CT showing both ends of the divided azygos vein (arrow). There is no postoperative failure due to the division of the azygos vein for 24 months postoperatively

Surgeons should be aware that thoracic surgical procedures performed in cases with an azygos lobe are associated with a high risk of complications. When performing thoracic procedures for lung cancer, invasion or adhesions to the mediastinum, azygos vein mesentery, or the azygos vein could pose a problem. Similarly, surgeons should also bear in mind the possibility of severe adhesions due to the azygos lobe during thoracoscopic surgery [11]. Conversion to open thoracotomy should be considered when the risk of serious intraoperative injuries appears to be high due to severe adhesions. Previous report indicated the risk of intraoperative azygos vein injury during thoracoscopic surgery in patients with azygos lobe [12]. Although the prone position is considered unsuitable for emergency thoracotomy, we obtained the position by bed rotation from the semi-prone position, which allows us to immediately change positions to the lateral decubitus position in case of emergency. In this study, we report a case of esophageal cancer with an azygos lobe successfully treated by thoracoscopic surgery. There have been surgical reports of lung cancer and pneumothorax coexisting the azygos lobe, and den Boer et al. reported a case of esophageal cancer with azygos lobe [13]. They tried robot-assisted minimally invasive esophagectomy after neoadjuvant chemoradiotherapy, however mediastinal lymph node dissection was limited due to the anatomical situation. To the best of our knowledge, this may be the first case in which radical mediastinal lymph node dissection was achieved during McKeown esophagectomy in patient with an azygos lobe. There was a possibility that the apex of the lung have also adherent due to the previous history of bacterial pyothorax in this case, and it was important to diagnose the presence of the azygos lobe in advance, and plan to divid the mesentery and the azygos vein which allowed us to complete the thoracoscopic esophagectomy without any injury or massive bleeding. The mesentery contiguous to the arch of the azygos vein was incised before the azygos vein was divided, and the superior mediastinum was dissected after the azygos vein was divided, which did not interfere with the surgical technique. There have been no reports of postoperative failure due to the division of the azygos vein in the patients with normal cardiac function. The clinical course in the present case has also remained uneventful, including respiratory and circulatory symptoms. for 36 months postoperatively. (Fig. 6) Regarding the approach to the esophagus, thoracoscopic approach is used to be performed for thoracic esophageal cancer as minimally invasive approach. On the other hand, recent technical advances in mediastinoscopic esophagectomy have been remarkable, therefore mediastinoscopic esophagectomy may be useful in cases with anatomic variations outside the mediastinum and be able to preserve azygos lobe.

In conclusion, preoperative recognition of the azygos lobe may help in preventing intraoperative injury. The probability of encountering an azygos lobe is very low and may not be well recognized. However, it is easy to diagnose preoperatively, and surgeons are required to try and rule out its presence prior to surgery to avoid the risk of serious intraoperative injury.

## Data Availability

The data supporting the findings of this study are available on request from the correspondence author and are not available publicly due to privacy of the patient or ethical restrictions.

## Abbreviations

CT: Computed tomography
MRI: Magnetic Resonance Imaging
